# COVID Vaccine Rollout for Older Adults in the United Kingdom

**DOI:** 10.1093/geroni/igab046.716

**Published:** 2021-12-17

**Authors:** Huajie Jin

**Affiliations:** King's College London, London, England, United Kingdom

## Abstract

As of early March, at least 22 million adults had received one dose of a Covid vaccine in the UK, with 1.2 million of those fully vaccinated with two shots. Anybody aged 56 and over can book an appointment to get the Covid-19 vaccine. We will provide detailed information later. The symposium has experts from 8 countries. We will use an interview and dialog format, instead of presentations.

